# Concurrent Heroin Use and Correlates among Methadone Maintenance Treatment Clients: A 12-Month Follow-up Study in Guangdong Province, China

**DOI:** 10.3390/ijerph13030305

**Published:** 2016-03-09

**Authors:** Xiaofeng Luo, Peizhen Zhao, Xiao Gong, Lei Zhang, Weiming Tang, Xia Zou, Wen Chen, Li Ling

**Affiliations:** 1 Faculty of Medical Statistics and Epidemiology, School of Public Health, Sun Yat-sen University, 74 Zhongshan Road II, Guangzhou 510080, China; luoxiaof@lzu.edu.cn (X.L.); x.gong@foxmail.com (X.G.); zouxia@mail3.sysu.edu.cn (X.Z.); chenw43@mail.sysu.edu.cn (W.C.); 2 Sun Yat-sen Center for Migrant Health Policy, Sun Yat-sen University, 74 Zhongshan Road II, Guangzhou 510080, China; 3 Guangdong Center for Skin Guangdong Provincial Center for Skin Diseases and STIs Control, No. 2 Lujing Road, Guangzhou 510091, China; 08tjzpz@163.com (P.Z.); weiming_tang@med.unc.edu (W.T.); 4 The Kirby Institute, University of New South Wales, Sydney NSW 2052, Australia; lei.zhang1@monash.edu

**Keywords:** concurrent drug use, Methadone Maintenance Treatment (MMT), China

## Abstract

*Objective*: To assess concurrent heroin use and correlates among Methadone Maintenance Treatment (MMT) clients in Guangdong Province, China. *Method*: Demographic and drug use data were collected with a structured questionnaire, and MMT information was obtained from the MMT clinic registration system in Guangdong. Human immunodeficiency virus (HIV-) and hepatitis C virus (HCV) infected status and urine morphine results were obtained from laboratory tests. Logistic regressions were employed to investigate the factors associated with concurrent heroin use. *Results*: Among the 6848 participants, 75% continued using heroin more than once during the first 12 months after treatment initiation. Concurrent heroin use was associated with inharmonious family relationship (OR (odds ratio) = 1.49, 95% CI (confidence intervals): 1.24–1.78), HIV positivity (OR = 1.25, 95% CI: 1.01–1.55), having multiple sex partners (OR = 1.34, 95% CI: 1.07–1.69), having ever taken intravenous drugs (OR = 0.81, 95% CI: 0.69–0.95), higher maintenance dose (OR = 1.13, 95% CI: 1.01–1.28) and poorer MMT attendance (OR_<20%_ = 1.32, 95% CI: 1.13–1.53; OR_20%–_ = 1.33, 95% CI: 1.14–1.54; OR_50%–_ = 1.69, 95% CI: 1.44–2.00). Among those who used heroin concurrently, the same factors, and additionally being older (OR_35–_ = 1.26, 95% CI: 1.11–1.43; OR_≥45_ = 1.63, 95% CI: 1.30–2.05) and female (OR = 1.60, 95% CI: 1.28–2.00), contribute to a greater frequency of heroin use. *Conclusions*: Concurrent heroin use was prevalent among MMT participants in Guangdong, underscoring the urgent needs for tailored interventions and health education programs for this population.

## 1. Introduction

China is adjacent to the “Golden Triangle” and “Golden Crescent”, two of the three largest drug production and manufacturing regions in the world, and has seen a substantial increase in the number of illicit drug users since the 1980s [1]. At the end of 2014, there were cumulatively 2.95 million drug users and 480,000 newly registered drug users nationwide (In China, public security organisms require drug users to register. This included those who were registered spontaneously, newly discovered, voluntary and compulsory abstainers and Methadone Maintenance Treatment (MMT) participants, and the data were recorded into a national unified MMT management system.) [2]. Drug use, particularly intravenous drug use (IDU), has contributed to the spread of the HIV epidemic in China, especially during the early onset of the epidemic [3].

Methadone Maintenance Treatment (MMT) has been well-accepted and recognized [4,5] due to its effectiveness in reducing opioid use [5], drug-related criminal activities [6], mortality [7], and HIV transmission [8,9], as well as facilitating the success of antiretroviral therapy for HIV-infected patients [10]. It has also been shown to improve the social well-being of drug users [11]. To address the growing IDU-driven HIV epidemic, China began operating the MMT program in 2004 as a pilot project [12], serving primarily as a preventive measures for controlling the spread of HIV among drug users and reducing opioid dependence among drug addicts. With the success of the pilot clinics, the program was formally launched in 2006 and as of April 2015, there are 767 clinics in 28 provinces (including autonomous regions and municipalities) across the nation. They provide maintenance treatment services for nearly 190,000 clients [13].

However, many clients never fully abstain from using opiates or other drugs while in treatment. Concurrent heroin use was a common phenomenon among MMT clients [14,15,16], and it subsequently exposed them to a greater risk. Yet, the MMT program providers often preferred to ignore this because ceasing treatment for continued drug use is associated with poorer client outcomes (in China, there is no specific regulation to deal with concurrent drug use of MMT clients.) [17]. Studies have shown that longer length of treatment is associated with the increased concurrent heroin use [15], and that concurrent heroin use would directly increase the drop-out rates from the MMT program [18].

The existing literature on concurrent heroin use among MMT clients are mainly based on self-reported data, one-time urine tests or fixed-cycle urine tests [14,15,16]. Currently, limited data are available based on consecutive and random urine tests in China. Consecutive and random urine tests could provide a greater window of opportunity for MMT clients to relapse into heroin use. In addition, previous studies have failed to assess the frequency of heroin use and correlates of greater-frequency heroin use over a 12-month period. Therefore, we designed and conducted this study in Guangdong Province to examine the rate and associated factors of concurrent heroin use, and the higher frequency of heroin use among concurrent heroin users. The study area was chosen for the following reasons: First, Guangdong is located on the Southern coast of China, the frontier of China’s economic reform and opening and also is the forerunner in terms of the HIV/AIDS epidemic; the total number of cumulative reported HIV-infected individuals was 48,718 as of October 2014, ranking fifth among provinces in China. According to estimation, there were approximately 72,000 people living with HIV/AIDS by October 2014 in Guangdong [19]. Furthermore, Guangdong has the largest number of registered drug users among all Chinese provinces, accounting for one-sixth (457,000) of the total of 2,760,000 registered drug users of all categories in China [20,21]. Currently, Guangdong has 62 established MMT clinics, currently providing services for 6517 clients (Guangdong Health and Family Planning Commission).

## 2. Experimental Section

### 2.1. Ethics Statement

Written informed consent was obtained from all study participants. The study protocol was reviewed and approved by the Institutional Review Board (IRB) of the School of Public Health, Sun Yat-sen University, Guangzhou, China (No: 2013-26).

### 2.2. Study Site and Participants

This study was conducted in 14 MMT clinics located in nine cities of Guangdong Province on the Southern coast of China. A roster of registered MMT clients in these clinics between July 2006 and March 2014 was obtained from the clinics management system as a sampling frame.

The study period (the duration we observed whether a dropout occurred) was defined as either the duration between enrolment and dropout, or the duration between enrolment and the end of the first 12 months after treatment initiation. Participants were eligible if they: (1) were able to provide informed consent; (2) were 18 years or older; (3) met the Chinese Classification of Mental Disorders version 3 criteria for opioid dependence and had no other serious mental disorders; (4) had not received MMT treatment in the last six months; (5) had no contraindications for taking methadone, and (6) had received MMT for more than one month and had completed at least one follow-up test (the first month was defined as the “introduction period”, many clients would continue to use drugs for transitional and insufficient methadone dose).

### 2.3. Study Procedure

At admission, each participant completed a face-to-face interview administered by a trained local public health worker (long-term practice and experience in MMT clinic helped them build the participants’ trust and subsequently obtained real self-report data.) in a separate or private setting (e.g., consulting room of clinic). The primary variables of interest included demographic characteristics, drug use and sexual activity over the past 3 months. Urine-based morphine tests were performed on a random day each month during the study period. Information on daily methadone doses and attendance were routinely collected and stored in the national unified MMT management system.

### 2.4. Measures

#### 2.4.1. Demographic Characteristics at Baseline

Demographic characteristics included age, gender, marital status, education level, employment status and family relationship.

#### 2.4.2. Multiple Sex Partners at Baseline (over the Past Three Months)

Multiple sex partner relationship was defined as one who had had sex with multiple partners over the past three months.

#### 2.4.3. Drug Use at Baseline

Duration of drug use was defined as the difference between registered age and the initial age at which participants first used either opium or heroin. One question in the questionnaire was used to measure the status of intravenous drugs: “Have you injected drugs since drug use initiation?” If a participant answered “yes” to this question, then he/she was further asked to report whether he/she had ever shared any of their injection equipment to others.

#### 2.4.4. Methadone Information (Maintenance Dose and Attendance)

Clients attend the clinics daily to obtain their methadone dose, the data of which were routinely collected and stored in the national unified MMT management system. Daily dose information was abstracted, and the average maintenance dose for the duration of treatment was calculated. The percentage of MMT attendance was calculated based on the number of days a patient received a dose over the study period.

#### 2.4.5. Concurrent Heroin Use during Study Period

At the study site, as always, opioid drugs including synthetic opioids were common, with powdered heroin being the most commonly abused. Most users administered opioids either by snorting or injection. Once in the body, the opioid metabolize into morphing.

Concurrent heroin use was based on the positive morphine urine results during the 12-month follow-up. Positive morphine urine results generally indicate heroin use by the participants within a few days. Urine morphine tests were performed on a random day each month during the study period. The percentage of heroin use was calculated based on the number of the positive urine morphine test results and the number of testing times.

According to the results of the urine morphine tests, the participants are currently grouped as “heroin use” and “no heroin use”. In addition, they are classified as <20%, 20%–80% and >80% based on the positive percentage of urine morphine tests.

#### 2.4.6. Laboratory Tests

##### HIV and HCV Tests

All participants except for those who were already confirmed as HIV-1/2- and/or HCV-infected before this study were invited, and agreed to receive HIV and/or HCV testing. Blood specimens were screened for HIV infection using an enzyme-linked immunosorbent assay (ELISA) technique (Beijing BGI-GBI Biotech Co., Ltd., Beijing, China). Any samples that screened positive for HIV were confirmed using a western blot assay (Abbott, MP Biomedicals, LLC, Singapore) by the local Centers for Disease Control. Meanwhile, blood samples from each client were tested for hepatitis C antibody using an enzyme-linked immunosorbent assay (ELISA) technique (ABON Biopharm Co., Ltd., Hangzhou, China). Pre- and post-test counseling services were provided, and if appropriate, national guidelines were followed in all counseling and testing services.

##### Urine Morphine Test

Each month, each participant was randomly chosen to test for morphine during the study period. Urine specimens were screened for morphine using a Morphine Diagnostic Kit (Colloidal Gold) technique (ABON Biopharm Co., Ltd., Hangzhou, China). All tests were performed according to the manufacturer’s instructions.

### 2.5. Statistical Analysis

Data were analyzed using SAS 9.2 for Windows (SAS Institute, Inc. Cary, NC, USA). In addition to descriptive analyses, tests of associations between two categorical variables were based on the chi-square test. Univariate and multivariate binary logistic regression analyses were performed to explore correlates of heroin use among the participants during the study period. Multivariate ordinal logistic regression analysis was conducted to explore correlates of higher percentage of heroin use among the participants during the study period. Results were reported as an odds ratio (OR) with 95% confidence intervals (CI) for the association between predictor variables. A significance level of 0.05 was used for all tests.

## 3. Results

### 3.1. Socio-Demographic Characteristics

In total, 6848 participants were enrolled in this study and the mean age was 35.9 (SD = 6.4). These participants were predominantly males, currently unmarried, had junior high school education, were unemployed and had harmonious family relationship, with proportions of 92.1%, 53.6%, 63.7%, 59.4% and 75.0% respectively (Table 1).

### 3.2. HIV and HCV Infection Status at Baseline

Within these participants, the prevalences of HIV infection and HCV infection were 8.0% and 76.8% respectively (Table 1).

### 3.3. Drug Use at Baseline

As shown in Table 1, 56.4% of the participants had used drugs for more than 10 years. Specifically, 54.7% of heroin users and 61.2% non-heroin users were at minimum 10-year drug users. Furthermore, over 66% had ever injected drugs and 4.2% of them had shared needles during the past 3 months (Table 1).

### 3.4. Average Maintenance Dose and Attendance

Among all participants, the average maintenance dose was (52.2 ± 22.5) mL, and roughly 70% of participants had received doses below 60 mL. In addition, only 25.6% participants’ attendance rates were over 80% (Table 1).

### 3.5. Heroin Use during the Study Period

Table 2 presents detailed information about heroin use among participants. Only 25.4% participants never used heroin during the study period. 23% participants were tested to have exceeded 80% positive outcomes on urine morphine tests.

Univariate binary logistic regression analysis indicated that being employed, being in an inharmonious family relationship, being HIV-positive, having a shorter duration of drug use, having ever injected drugs and having a poorer percentage of MMT attendance were independently associated with concurrent heroin use. After controlling for potential confounding variables, multivariate binary logistic regression analysis indicated that concurrent heroin use was independently associated with bad family relationships (OR = 1.49, 95% CI: 1.24–1.78), being HIV-positive (OR = 1.25, 95% CI: 1.01–1.55), having multiple sex partners at baseline (OR = 1.34, 95% CI: 1.07–1.69), having ever used intravenous drugs (OR = 0.81, 95% CI: 0.69–0.95), more maintenance doses (OR = 1.13, 95% CI: 1.01–1.28) and a poorer percentage of MMT attendance (OR_<20%_ = 1.32, 95% CI: 1.13–1.53; OR_20%__–_ = 1.33, 95% CI: 1.14–1.54; OR_50%__–_ = 1.69, 95% CI: 1.44–2.00). However, it was not significantly associated with age, gender, marital status, education, HCV infection status at baseline and duration of drug use (Table 3).

Multivariate ordinal logistic regression analysis indicated that after controlling for potential confounding variables among the concurrent heroin users, a higher percentage of concurrent heroin use was independently associated with the elderly (OR_35-_ = 1.26, 95% CI: 1.11–1.43; OR_≥45_ = 1.63, 95% CI: 1.30–2.05), female (OR = 1.60, 95% CI: 1.28–2.00), bad family relationships (OR = 1.56, 95% CI: 1.31–1.87), HIV-positive (OR = 1.76, 95% CI: 1.42–2.19), never used intravenous drugs (OR = 1.29, 95% CI: 1.10–1.52) and a poorer percentage of MMT attendance (OR_<20%_ = 38.43, 95% CI: 31.60–46.74; OR_20%__–_ = 5.42, 95% CI: 4.57–6.44; OR_50%__–_ = 2.11, 95% CI: 1.77–2.49). However, it was not significantly associated with marital status, education level, employment status, HCV infection status, multiple sex partners, duration of drug abuse and maintenance dose (Table 4).

## 4. Discussion

Concurrent heroin use among MMT clients is a long-standing and broadly recognized phenomenon worldwide [15]. This, to the best of our knowledge, is the only large sample, long-term consecutive study specifically designed to examine concurrent heroin use among MMT clients in Guangdong province. We revealed that the overall rate of heroin use among MMT clients in Guangdong was 74.6%, a percentage exceeding that of previous studies in China [14,15,16]. Sullivan *et al.* [14] reported that the rates of positive urine opiate tests and self-reported drug use in the last month of their study were 28.1 and 24.9 respectively. Li *et al.* [15] have shown that 44.9% of their clients either reported illicit heroin use over the past 30 days or had had a positive urine test. Wang *et al.* [16] found that the illicit heroin use rate was 10.4% based on urine morphine tests. This may be due to the fact that: (1) our results were obtained based on a 12-month period of consecutive urine tests; or (2) the urine specimens were collected monthly on a random day, which was blind to clients hoping to deliberately avoid the test time. Given that concurrent heroin use might be significantly associated with the retention in MMT [18] and health-related quality of life [10], targeted intervention programs are urgently needed for those clients in the study.

We observed that the factors with strong positive association with concurrent heroin use and/or a higher percentage of heroin use were: age, gender and HIV-infected status. The elderly generally had long durations of drug abuse, and demonstrated more dependence on drugs. HIV-positive individuals were more likely to use heroin and had a higher use frequency, which might have been due to this subgroup being required to take a higher methadone dose discussed in detail below [22]. The elderly and females generally had their own families, which required them to spend much energy and time on household work, sometimes causing them to miss the clinic time. Additionally, due to belief of Chinese culture, the elderly and females often feared revealing their identity, and the subsequent significant stigma and discrimination. This likely impeded their attendance to MMT [23,24].

Meanwhile, multiple sex partner behavior was positively associated with continued heroin use. Those clients, who had unstable family and social statuses, together with peer pressure and enticement from sexual partners who generally were drug users, showed greater likelihood of continued heroin use [25]. An interesting finding was: compared to IDUs, non-IDUs were more likely to both use heroin and to frequently use heroin. This may have been because methadone doses prescribed to IDUs was appropriate, however this speculation requires further investigation. The variable “duration of drug use” was not statistically significant in multivariate analysis, possibly due to the processes of recovery and relapse over time (that is, duration is not fixed; it fluctuates over time and is influenced by periods of recovery and relapse).

For many drug users, drug use often leaves them abandoned by their family members and non-drug-using friends. The influence of drug-using friends might substantially attribute to initiation or continuation of drug use in drug addicts [26]. Family relationships play a crucial role in drug recovery, and harmonious relationship could potentially encourage treatment participation and compliance [23]. Therefore it is vital to involve their families in MMT to foster their understanding of treatment goals. It is also crucial to help clients establish new social networks so as to keep them away from their existing drug-using friends, and to help them reintegrate into society.

The dose of MMT is an important factor influencing retention in methadone treatment, and several systematic reviews and randomized control trials have reported that higher doses of MMT are associated with longer retention [27,28,29]. A notable finding in this study was that the clients who received higher MMT doses were more likely to use illicit drugs, which contradicts with previous studies [16,27]. This might occur because of lower daily average maintenance doses in this study compared with that of prior study (52.23 mL *versus* 68.8 mL) [16]. In China, both staff and clients have a preference for lower doses [30], however occasionally this did not have the intended effect. Literature has shown that MMT clients in China receive lower doses of methadone compared with clients treated in other countries [4,31]. We speculated that (1) lower doses compared with what had been suggested in other counties may contribute to concurrent heroin use during MMT in China; (2) it is possible that those clients with less severe addiction were able to do well in treatment on low doses, while those in need of a higher dose were unable to remain in MMT; (3) among the study participants, 59.4% were unemployed, and for those who were employed, many were working short-term jobs. Expansive expenditures on drug use might have made it so that the participants with higher methadone dosage levels had to retain in the MMT to avoid withdrawal symptoms. For the participants with lower methadone doses levels, there were lower costs, which might have made them more likely to drop out and fail to be followed up; in addition, if clients with higher doses had poor attendance and frequently missed their daily methadone doses, they may have been more likely to use heroin to avoid withdrawal symptoms than the clients who had remained on lower doses. Further research is needed to verify whether increasing doses could decrease the incidence of concurrent heroin use.

Additionally, we found that poorer attendance was associated with both concurrent heroin use and higher percentage of heroin use, which were consistent with previous studies in other areas of China [14]. Methadone’s half-life is approximately 24 h, and MMT is a safe, corrective substitute treatment that requires long-term or even life-long intake of methadone at an adequate dose and on a daily basis [32]. Poorer attendance paves the way for concurrent heroin use. Therefore, maintaining higher attendance in treatment is critical to achieving better outcomes.

This study is subject to certain limitations. Firstly, behavioral information at baseline was self-reported and information about drug use and sexual activity was sensitive. Thus recall bias and deliberate concealment might be unavoidable. Secondly, some individuals with heroin use refused to get tested, and even quit MMT for this reason. Therefore, the drop-out incidences of this subgroup lead to underestimation. Thirdly, the urine morphine test merely indicated drug use within a few days. In our study, monthly urine tests were performed instead of weekly tests, which could have potentially lowered the rate of positive urine tests. In addition, China is experiencing a significant increase in the use of new drugs, with methamphetamine (MAMP) being the most commonly abused. It is the second most used drug after heroin [33]. This study did not focus on methamphetamine use among MMT clients.

## 5. Conclusions

Findings from this study provide important implications for future harm reduction programs targeting heroin use among clients on MMT adherence in Guangdong. A study conducted at the same site showed that misconceptions might predict dropout rates and poor adherence among newly admitted first-time methadone maintenance treatment clients [34]. Given all the above, first, continuous and efficient MMT consulting and health education interventions need to be conducted for reducing continued heroin use, especially for those who have sub-par family relationship, are HIV-infected, have multiple sex partners or have ever used intravenous drugs at baseline. Also, target programs involved the elderly and the females should be strengthened. Second, given that those who took higher doses and had poorer attendance are crucial groups during the MMT course, we recommend (1) future research should look to evaluate the effectiveness of higher-dose MMT *vs.* the standard dose; (2) social and family support and supervision measures should be strengthened in order to potentially improve MMT attendance. In addition, national education programs should be launched to reduce drug-use stigma in China.

## Figures and Tables

**Table 1:** Characteristics of study participants (*n* = 6848).

Variables	Heroin Use (*n* = 5110)	No Heroin Use (*n* = 1738)	Total (*n* = 6848)
	**No. (%)**	**No. (%)**	**No. (%)**
Age (years) (χ^2^ = 0.07, *p =* 0.964)			
18–	2384 (46.5)	808 (46.5)	3192 (46.6)
35–	2310 (45.2)	785 (45.2)	3095 (45.2)
≥45	416 (8.3)	145 (8.3)	561 (8.2)
Gender (χ^2^ = 3.02, *p* = 0.082 )			
Male	4721 (92.4)	1583 (91.1)	6304 (92.1)
Female	389 (7.6)	155 (8.9)	544 (7.9)
Marital Status (χ^2^ = 2.76, *p* = 0.097)			
Married Currently	2402 (47.0)	777 (44.7)	3179 (46.4)
Other	2708 (53.0)	961 (55.3)	3669 (53.6)
Education Level (χ^2^ = 0.53, *p* = 0.768)			
Illiterate or primary school	992 (19.4)	327 (18.8)	1319 (19.3)
Junior high school	3244 (63.5)	1120 (64.4)	4364 (63.7)
Senior high school	874 (17.1)	291 (16.7)	1165 (17.0)
Employment Status (**χ^2^ = 7.77, *p* = 0.005**)			
Employed	2126 (41.6)	657 (37.8)	2783 (40.6)
Unemployed	2984 (58.4)	1081 (62.2)	4065 (59.4)
Family Relationship (**χ^2^ = 64.45, *p* < 0.001**)			
Harmonious	3705 (72.5)	1428 (82.2)	5133 (75.0)
Inharmonious	1405 (27.5)	310 (17.8)	1715 (25.0)
HIV-Infected at Baseline (**χ^2^ = 5.92, *p* = 0.015**)			
Yes	435 (8.5)	116 (6.7)	551 (8.0)
No	4675 (91.5)	1622 (93.3)	6297 (92.0)
HCV-Infected at Baseline (χ^2^ = 2.35, *p* = 0.126)			
Yes	3901 (76.3)	1358 (78.1)	5259 (76.8)
No	1209 (23.7)	380 (21.9)	1589 (23.2)
Multiple Sex Partners at Baseline (χ^2^ = 2.68 , *p* = 0.102)			
Yes	358 (7.0)	102 (5.9)	460 (6.7)
No	4752 (93.0)	1636 (94.1)	6388 (93.3)
Duration of Drug Use (years) (**χ^2^** = **44.02, *p* < 0.001**)			
<5	1485 (29.1)	363 (20.9)	1848 (27.0)
5–10	829 (16.2)	311 (17.9)	1140 (16.6)
≥10	2796 (54.7)	1064 (61.2)	3860 (56.4)
Intravenous Drug Use at Baseline (**χ^2^ = 47.36, *p* < 0.001**)			
Yes	3238 (63.4)	1259 (72.4)	4497 (65.7)
No	1872 (36.6)	479 (27.6)	2351 (34.3)
Shared Intravenous Needles at Baseline (**χ^2^ = 18.15, *p <* 0.001**)			
Yes	238 (4.7)	49 (2.8)	287 (4.2)
No	3000 (95.3)	1210 (97.2)	4210 (95.8)
Average Maintenance Doses (mL) (χ^2^ = 3.37 , *p* = 0.066)			
<60	3553 (69.5)	1249 (71.9)	4802 (70.1)
≥60	1557 (30.5)	489 (28.1)	2046 (29.9)
Percentage of MMT Attendance (%) (**χ^2^ = 44.02, *p* < 0.001**)			
<20	1327 (26.0)	442 (25.4)	1769 (25.8)
20–	1411 (27.6)	467 (26.9)	1878 (27.4)
50–	1155 (22.6)	295 (17.0)	1450 (21.2)
≥80%	1217 (23.8)	534 (30.7)	1751 (25.6)

**Table 2:** The positive percentages of urine morphine testing results among participants during the study period (*n* = 6848).

Positive Percentage (%)	Number (*n*)	Percentage (%)
0	1738	25.38
>0	311	4.54
10–	721	10.53
20–	478	6.98
30–	532	7.77
40–	671	9.80
50–	233	3.40
60–	325	4.75
70–	318	4.64
80–	154	2.25
90–	1367	19.96
Total	6848	100.0

**Table 3:** Correlates of concurrent heroin use among the participants during study period (*n* = 6848).

Variables	Univariate		Multivariate	
	OR (95% CI) ^a^	*p*	OR (95% CI)	*p*
Age (years)				
18–	1.03 (0.84–1.26)	0.789	0.93 (0.75–1.16)	0.534
35–	1.03 (0.83–1.31)	0.809	0.98 (0.79–1.21)	0.843
≥45	1.00		1.00	
Gender				
Male	1.19 (0.98–1.44)	0.082	1.14 (0.93–1.39)	0.199
Female	1.00		1.00	
Marital Status				
Married Currently	1.10 (0.98–1.22)	0.097	1.10 (0.98–1.24)	0.093
Others	1.00		1.00	
Education Level				
Illiterate or primary school	1.01 (0.84–1.21)	0.914	0.98 (0.82–1.19)	0.867
Junior high school	0.96 (0.83–0.12)	0.633	0.95 (0.82–1.11)	0.513
Senior high school	1.00		1.00	
Employment Status				
Employed	1.17 (1.05–1.31)	**0.005**	0.92 (0.82–1.03)	0.146
Unemployed	1.00		1.00	
Family Relationship				
Harmonious	1.00		1.00	
Inharmonious	1.75 (1.52–2.00)	**<0.001**	1.49 (1.24–1.78)	**<0.001**
HIV-Infected at Baseline				
Yes	1.30 (1.05–1.61)	**0.015**	1.25 (1.01–1.55)	**0.047**
No	1.00		1.00	
HCV-Infected at Baseline				
Yes	0.90 (0.79–1.03)	0.126	1.08 (0.93–1.24)	0.324
No	1.00		1.00	
Multiple Sex Partners at Baseline				
Yes	1.21 (0.96–1.52)	0.102	1.34 (1.07–1.69)	**0.012**
No	1.00		1.00	
Duration of Drug Use (years)				
<5	1.56 (1.36–1.78)	**<0.001**	1.10 (0.91–1.33)	0.347
5–10	1.01 (0.88–1.18)	0.850	1.04 (0.89–1.21)	0.658
≥10	1.00		1.00	
Intravenous Drug Use at Baseline				
Yes	0.66 (0.58–0.74)	**<0.001**	0.81 (0.69–0.95)	**0.009**
No	1.00		1.00	
Average Maintenance Doses (mL)				
<60	1.00		1.00	
≥60	1.12 (0.99–1.26)	0.066	1.13 (1.01–1.28)	**0.047**
Percentage of MMT Attendance (%)				
<20	1.32 (1.14–1.53)	**<0.001**	1.32 (1.13–1.53)	**<0.001**
20–	1.33 (1.15–1.53)	**<0.001**	1.33 (1.14–1.54)	**<0.001**
50–	1.72 (1.46–2.02)	**<0.001**	1.69 (1.44–2.00)	**<0.001**
≥80%	1.00		1.00	

**^a^** OR: Odds Ratio; CI: Confidence Interval, obtained from binary logistic regression analysis.

**Table 4:** Correlates of higher percentage of heroin use among the concurrent heroin users during study period (*n* = 5110).

Variables	<20% *n* (%) *	20%–80% *n* (%) *	>80% *n* (%) *	OR (95% CI) ^a,b^	*p* ^b^
Age (years)					
18–	378 (15.86)	1319 (55.33)	687 (28.82)	1.00	
35–	357 (15.45)	1264 (54.72)	689 (29.83)	1.26 (1.11–1.43)	**<0.001**
≥45	67 (16.11)	204 (49.04)	145 (34.86)	1.63 (1.30–2.05)	**<0.001**
Gender					
Male	737 (15.61)	2587 (54.80)	1397 (29.59)	1.00	
Female	65 (16.71)	200 (51.41)	124 (31.88)	1.60 (1.28–2.00)	**<0.001**
Marital Status					
Married Currently	379 (15.78)	1322 (55.04)	701 (29.18)	0.95 (0.85–1.07)	0.441
Others	423 (15.62)	1465 (54.10)	820 (30.28)	1.00	
Education Level					****
Illiterate or primary school	169 (17.04)	521 (52.52)	302 (30.44)	0.86 (0.71–1.04)	0.116
Junior high school	490 (15.10)	1764 (54.38)	990 (30.52)	0.97 (0.83–1.14)	0.710
Senior high school	143 (16.36)	502 (57.44)	229 (26.20)	1.00	
Employed Status					
Employed	302 (14.21)	1121 (52.73)	703 (33.07)	1.00	****
Unemployed	500 (16.76)	1666 (55.83)	818 (27.41)	1.02 (0.91–1.15)	0.717
Family Relationship					
Harmonious	222 (16.64)	752 (56.37)	360 (26.99)	1.00	
Inharmonious	133 (9.47)	760 (54.09)	512 (36.44)	1.56 (1.31–1.87)	**<0.001**
HIV-Infected at Baseline					
Yes	35 (8.05)	243 (55.86)	157 (36.09)	1.76 (1.42–2.19)	**<0.001**
No	767 (16.41)	2544 (54.42)	1364 (29.18)	1.00	
HCV-Infected at Baseline					
Yes	658(16.87)	2176(55.78)	1067(27.35)	0.91(0.79–1.06)	0.234
No	144(11.91)	611(50.54)	454(37.55)	1.00	
Multiple Sex Partners at Baseline					
Yes	736 (15.49)	2612 (54.97)	1404 (29.55)	0.95 (0.76–1.19)	0.655
No	66 (18.44)	175 (48.88)	117 (32.68)	1.00	
Duration of Drug Use (years)					
<5	143 (9.63)	808 (54.41)	534 (35.96)	1.21 (0.99–1.47)	0.062
5–10	147 (17.73)	436 (52.59)	246 (29.67)	1.17 (0.99–1.39)	0.063
≥10	512 (18.31)	1543 (55.19)	741 (26.50)	1.00	
Intravenous Drug Use at Baseline					
Yes	592 (18.28)	1798 (55.53)	848 (26.19)	1.00	
No	210 (11.22)	989 (52.83)	673 (35.95)	1.29 (1.10–1.52)	**0.002**
Average Maintenance Doses (mL)					****
<60	550 (15.48)	1882 (52.97)	1121 (31.55)	1.00	****
≥60	252 (16.18)	905 (58.12)	400 (25.69)	1.19 (0.98–1.27)	0.085
Percentage of MMT Attendance (%)					****
<20	20 (1.51)	382 (28.79)	925 (69.71)	38.43 (31.60–46.74)	**<0.001**
20–	117 (8.29)	936 (66.34)	358 (25.37)	5.42 (4.57–6.44)	**<0.001**
50–	233 (20.17)	777 (67.27)	145 (12.55)	2.11 (1.77–2.49)	**<0.001**
≥80%	432 (35.50)	692 (58.86)	93 (7.64)	1.00	****

^a^ OR: Odds Ratio, CI: Confidence Interval; ^b^ Obtained from multivariate ordinal logistic regression analysis adjusting for potential confounding variables listed in the table; * Proportions were calculated in the row.
